# Older Adults’ Trust and Distrust in COVID-19 Public Health Information: Qualitative Critical Incident Study

**DOI:** 10.2196/42517

**Published:** 2023-11-09

**Authors:** Kristina Shiroma, Tara Zimmerman, Bo Xie, Kenneth R Fleischmann, Kate Rich, Min Kyung Lee, Nitin Verma, Chenyan Jia

**Affiliations:** 1 School of Information The University of Texas at Austin Austin, TX United States; 2 School of Nursing The University of Texas at Austin Austin, TX United States; 3 Department of Communication The University of Washington Seattle, WA United States; 4 School of Journalism The University of Texas at Austin Austin, TX United States

**Keywords:** health information, information-seeking behavior, COVID-19, qualitative research methods, communication, media and networks, aging, older adults, elderly population, mass media, public health information, trust

## Abstract

**Background:**

The COVID-19 infodemic has imposed a disproportionate burden on older adults who face increased challenges in accessing and assessing public health information, but little is known about factors influencing older adults’ trust in public health information during COVID-19.

**Objective:**

This study aims to identify sources that older adults turn to for trusted COVID-19 public health information and factors that influence their trust. In addition, we explore the relationship between public health information sources and trust factors.

**Methods:**

Adults aged 65 years or older (N=30; mean age 71.6, SD 5.57; range 65-84 years) were recruited using Prime Panels. Semistructured phone interviews, guided by critical incident technique, were conducted in October and November 2020. Participants were asked about their sources of COVID-19 public health information, the trustworthiness of that information, and factors influencing their trust. Interview data were examined with thematic analysis.

**Results:**

Mass media, known individuals, and the internet were the older adults’ main sources for COVID-19 public health information. Although they used social media for entertainment and personal communication, the older adults actively avoided accessing or sharing COVID-19 information on social media. Factors influencing their trust in COVID-19 public health information included confirmation bias, personal research, resigned acceptance, and personal relevance.

**Conclusions:**

These findings shed light on older adults’ use of information sources and their criteria for evaluating the trustworthiness of public health information during a pandemic. They have implications for the future development of effective public health communication, policies, and interventions for older adults during health crises.

## Introduction

Older adults have been disproportionately affected by the COVID-19 pandemic [1] and by the resulting infodemic of rapidly spreading misinformation and disinformation about it [2-4]. Finding credible, trustworthy public health information about COVID-19 can be daunting for anyone, but it has been especially difficult for older adults who tend to face challenges in using digital technology to access information and services [3]. Preliminary evidence suggests that older adults may be more susceptible to misinformation [5], and older adults can lack confidence in assessing the quality of health information on the web [6]. More research is needed in order to understand the sources and factors that influence older adults’ trust in public health information during a pandemic.

Compared with younger adults, who tend to rely on health information found on the internet [7,8], older adults are more likely to experience difficulties in sifting through the large quantities of health information on the web [9-11]. Older adults use social media to stay in touch with family and friends [12,13], and, increasingly, to locate or share health information as well [14]. Yet they still turn to health care providers as a foundational source for health information, and they still rely on direct personal contacts such as friends and family for help in obtaining and interpreting health information from web-based sources [15]. Researchers have identified differences in how older adults understand web-based information and how this impacts their trust in it [16]. For example, increased exposure to and repetition of information over time can increase trust in that information; this has been called a repetition-induced truth effect [16,17]. Similarly, how people choose to share health education is affected by the tendency to select information that aligns with established beliefs, or confirmation bias [18,19].

Uncertainty, fear, and social isolation associated with COVID-19 can not only hinder the integration of information from trusted sources but also increase one’s susceptibility to misinformation and disinformation [20,21]. During the pandemic, older adults may have felt overwhelmed by the quantity of information about COVID-19 available [3,22]. Whereas younger adults turn to social media platforms such as Facebook, Twitter, or YouTube for COVID-19 public health information, older adults often rely on traditional mass media such as television, radio, and newspapers [20], as well as on their personal contacts with family and friends [22,23].

Beyond accessing and assessing sources for COVID-19 public health information, people must also evaluate the information itself. One way to do so is to seek and compare information across multiple sources [24]. Older adults seem to be more likely than younger people to fact-check or research new information that they have heard about COVID-19, which includes drawing upon their previous life experiences to help determine the information’s trustworthiness [25]. Nevertheless, people across age groups tend to seek information that confirms their existing political beliefs [26], and the politicization of information about COVID-19 represents a danger to individuals’ willingness to fact-check information.

Personal criteria for assessing information’s trustworthiness can vary, but little is understood about older adults’ criteria for vetting COVID-19 public health information and the interplay among those criteria. Our multiphase research project, funded by the National Science Foundation, is intended to address these gaps in the literature. Here, we report findings from phase 2 of the study, in which we conducted semistructured in-depth interviews to further understand factors that might influence older adults’ trust in information sources as well as the information itself. Our research questions (RQs) were as follows:

1. RQ1: From what information sources do older adults seek, receive, evaluate, and use information about COVID-19?
2. RQ2: What factors might influence older adults’ trust in the COVID-19 information that they obtain from various information sources?

To answer our RQs, we used the critical incident technique (CIT) [27,28] to develop an interview guide for our interviews. As a qualitative interview method, the CIT allows participants to self-identify and reflect on significant events. We chose to use the CIT to inductively identify COVID-19 public health information sources and the factors that influenced older adults’ trust. At the time of the interviews, information about a possible COVID-19 vaccine was beginning to circulate in the media. Through the CIT, we were able to gain a snapshot of older adults’ perspectives of trust in COVID-19 public health information at a critical moment during the pandemic.

## Methods

### Design

In semistructured interviews, we used the CIT [27,28] to engage participants in recalling and discussing specific examples of COVID-19 public health information. For example, we asked participants to recall the most recent health information about COVID-19 that they had heard (see Multimedia Appendix 1).

### Participants and Recruitment

Participants were a purposive sample of 30 US older adults aged 65 to 84 (mean age 71.6, SD 5.57) years interviewed in October and November 2020. They were recruited from a pool of 123 older adults from phase 1 of our study who indicated a willingness to participate in a follow-up interview. We used Prime Panels for all recruitment. Of 123 older adults contacted, 48 (39%) responded. Those in our final sample of 30 were chosen to best reach a balance of political inclination (Democrat: n=11, 37%; Independent or other: n=8, 27%; and Republican: n=11, 37%), education (with degree: n=16, 53% and without degree: n=14, 47%), and self-identified gender (female: n=19, 63% and male: n=11, 37%). However, we were unable to balance participants on the basis of race or ethnicity (Black or African American: n=1, 3% and White: n=29, 97%).

### Materials and Measurements

A list of predetermined questions guided each interview (see Multimedia Appendix 1). The CIT informed the development of our interview guide, such that questions focused on the most recent COVID-19 public health information that participants had encountered as well as specific examples of COVID-19 public health information that they found trustworthy or untrustworthy. To begin each interview, we elicited their recollection of critical incidents by asking the following question: “From what source did you first learn about COVID-19?” Additional follow-up questions facilitated clarification and provided additional depth as needed to obtain insight into individuals’ personal motivations and to understand unique situations [29]. For example, after asking about specific examples of information that participants trusted or distrusted, we asked them “Why did you trust/distrust this information?” “What was the information source?” and “Has your trust/distrust in this information changed over time?”

### Ethical Considerations

A total of 2 team members conducted telephone interviews with each participant, with 1 serving as the interviewer and the other taking notes. At the start of each phone call, the interviewer obtained verbal consent to audio record the interview. At the beginning of the audio recording, the interviewer read a brief consent statement to the participant, answered any of the participant’s questions, and asked for verbal informed consent to proceed. The interviews were recorded with AISense, Inc’s Otter.ai transcription software, with Apple’s QuickTime as a backup. Each interview lasted approximately 1 hour. At the conclusion of each interview, the participant received a US $20 Amazon eGift card. This study’s procedures were approved by the institutional review board of The University of Texas at Austin (IRB study #2020-03-0080).

### Data Analysis

The interview transcriptions automatically generated by Otter.ai were reviewed and manually corrected as necessary by the interviewer and the notetaker to ensure accuracy. The interviewer then uploaded each transcription into Dedoose’s web-based qualitative data analysis software, and both the interviewer and the notetaker independently coded the data in Dedoose using inductive thematic analysis [30]. The goal was to identify themes or patterns in the participants’ responses that reflected their beliefs about COVID-19, as well as trusted and distrusted sources of public health information. First, we (the interviewer and the notetaker) familiarized ourselves with the interview data through multiple readings of the transcripts. In this step, both researchers noted initial ideas, highlighting passages in which participants spoke about COVID-19 public health information, trusted or distrusted sources of COVID-19 information, and personal impacts of the pandemic on their lives. Second, through iterative rereading and discussion, we developed initial codes for the interview data. For example, the code “information source” identified instances in which participants discussed using a specific resource for COVID-19 public health information. Next, we collated the coded data, assessed the data for themes, and developed initial themes; examples included “active avoidance of social media,” “human versus digital sources of information,” and “common sense as a factor in trust.” A third team member then reviewed the data as a separate coder, and the research team met as a group to discuss that coder’s findings and establish agreement on initial themes. We reviewed the initial themes in relation to both the coded extracts and the data set as a whole, and we discussed, revised, and finalized the themes as a team.

## Results

### Overview

The data revealed 2 main themes. The first theme, “Sources of COVID-19 Public Health Information,” represents sources to which participants turned for public health information about COVID-19. Under this theme, we identified 3 subthemes: mass media, known individuals, and the internet. The second theme, “Older Adults’ Criteria for Trusting COVID-19 Public Health Information,” represents the factors that participants discussed in assessing the trustworthiness of their public health information sources. Under this theme, we identified 4 subthemes: personal relevance, personal research, confirmation bias, and resigned acceptance.

### Sources of COVID-19 Public Health Information

#### Overview

The older adults in the study turned to mass media, individuals with whom they shared personal relationships (ie, known individuals), and the internet to access COVID-19 public health information. They turned to mass media not only for national news but also for local and regional information about COVID-19’s impact on their communities. Years of reliance on mass media were often given as a reason for trusting such sources to provide reliable COVID-19 public health information. The participants also placed trust in information obtained directly from known individuals, and often, they did not think it was necessary to verify such information. When participants did feel a need to verify COVID-19 public health information, they used the internet to fact-check it.

Although the older adults reported using social media to connect with others or for entertainment, they were adamant about not using social media to access important public health information about COVID-19. Of the 70% (21/30) of participants who said that they had used social media, 16 (53%) said that they avoided seeking COVID-19 public health information from social media. Participants found social media posts to be unverifiable and had a general distrust of using social media for health information. Some even considered social media to be sinister or dangerous.

Given the breadth of these 3 categories of information sources (mass media, known individuals, and the internet), we subsequently identified subcategories under each. Mass media included information sources such as television news, radio programs, podcasts, and newspapers. Known individuals included friends, family, and personal doctors. As for the internet, participants typically consulted websites maintained by authoritative health organizations or simply used search engines such as Google.

#### Mass Media

Participants used mass media such as television, newspapers, radio, and podcasts as sources of information. Some had a generalized trust in certain mass media sources: “Fox, NBC, CBS, any of them...I really don’t distrust them. Because they have to maintain a level of believability in anything to be believable in all things.” Others expressed preferences for specific cable networks or programs: “CNN, my best friend,” or “I love my 60 Minutes.” Some said that they accessed COVID-19 information from radio stations: “I do listen to conservative talk radio at least once a day.” Participants also discussed mass media personalities as trusted individuals. One participant discussed a favorite newscaster: “He [Don Lemon] just seems trustworthy; he seems to report it as it is.” Other trusted mass media personalities were medical experts such as Dr Sanjay Gupta: “Dr. Gupta from CNN...I like him when he’s on. I think he is excellent, and he presents everything objectively.”

#### Known Individuals

For trusted COVID-19 public health information, participants relied on people with whom they shared personal relationships, such as family, friends, and their own doctors. Family members were frequently mentioned: “My daughter follows something and gets alerts constantly on the phone, so she’s always updating me with everything.” Others spoke of their friends: “My ex-husband’s wife is a nurse and we’re good friends. And she says they are fudging the numbers.” Some received COVID-19 public health information directly from their doctors: “Well, this is from...not on a news source. I just happened to go for an annual checkup 2 days ago and just said to the doctor, ‘Hey, what’s going on with this?’”

#### Internet

Participants also searched the internet for information from websites maintained by authoritative health organizations. One referred to using these websites as a source for fact-checking: “If I had doubts, I would go to a website like...the World Health Organization website, CDC website, something like that.” Others did not recognize specific websites as information sources but instead spoke of search engines such as Google or Bing: “I go to Bing.com every morning...That’s where I get most of my stuff, on the internet.”

Participants discussed using the internet to verify information obtained from mass media: “At this point, when I hear something on CBS, I go out on the internet and see what the health organizations are saying about it, to back up what I’m hearing.” They also used the internet to verify the credentials of medical experts seen on mass media: **“**One or two [experts] I looked up on the internet just to confirm my own reasoning, to find out what their credentials were.”

Participants also used the internet to access and share COVID-19 public health information with individuals. This could include sharing information with one’s personal doctor: “[My doctor] was sort of fascinated by it too, so we went online and we were talking about the information.” One participant thought that the internet was where a health professional would go for information, so the internet must be a good source: “I think people in the medical field go to those websites [CDC]...I feel more comfortable in knowing the facts, and then I make my own decision.”

### Older Adults’ Criteria for Trusting COVID-19 Public Health Information

#### Overview

In assessing the trustworthiness of public health information about COVID-19, participants relied on 4 personal criteria: personal relevance, personal research, confirmation bias, and resigned acceptance. Each of these criteria was situated either endogenously (as an internal factor) or exogenously (as an outward factor). In addition, these personal criteria were used to assess trust in either the information itself or the information’s source.

**Figure 1:**
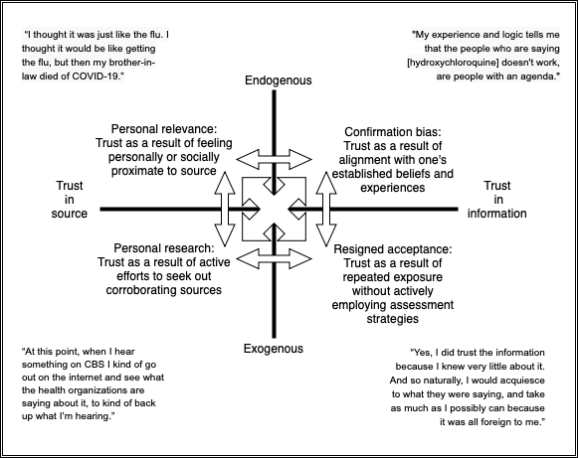
Personal criteria for trusting information.

The semiotic square in Figure 1 illustrates the relationships among these 4 criteria acting as factors, along with how they influenced participants’ trust in COVID-19 public health information. Each personal criterion is situated as an endogenous or exogenous factor as well as according to whether trust is attributed to the information source or to the information itself. Thus, Figure 1 illustrates relationships among opposing concepts. Personal relevance and research are based on trust in the information source, whereas confirmation bias and resigned acceptance are based on trust in the information itself. Personal relevance and confirmation bias are both endogenous, generated from within the individual; personal research and resigned acceptance are exogenous, depending primarily on information and experience external to the individual.

#### Personal Relevance

Personal relevance, the degree to which participants felt personally or socially proximate to an information source, was 1 criterion in trusting new COVID-19 public health information. Personal relevance is an endogenous criterion in which one uses one’s unique life experience to assess an information source’s trustworthiness.

Although international and national news may have been difficult to believe early on during the COVID-19 pandemic, as information grew personally or socially proximate, participants began to believe in it. State and local authorities were often mentioned as proximate trusted sources: “Our governor did come on TV every day at 2:30 pm for about a month straight to give us information about what we had to do as a state. And that was very, very helpful.” Similarly, another participant’s trust in the governor was based on what could be seen in the local community: “So far I have not heard anything from [the governor] that I would lose trust in because it seems to fit in with everything around my community.” Others expressed trust in local health authorities: “I trust everything that’s coming out of the Oregon Health Authority.”

The more local the information was, the more the participant trusted that information source: “I’ll get [my information] locally from the emergency management, see where they’re coming from. At least there are people there we can trust.” Another participant who resided in an assisted living facility said “[They] send memos and notes and emails from time to time. They provide a lot of information. It’s very helpful.” COVID-19 had to become socially proximate before some could trust the reported severity: “I thought it would be like getting the flu, but then my brother-in-law died of COVID-19.”

#### Personal Research

Another criterion that contributed to trust in COVID-19 public health information was personal research, which varied in style and level of detail. Personal research, or the active seeking of corroborating sources of information, was a frequent intentional strategy among the participants. Because personal research necessitates searching for information that reaches beyond one’s prior understanding, it is an exogenous criterion for assessing trust in information sources.

Some participants compared emerging COVID-19 information from 1 source with that from another: “Nothing they’ve said disagrees with everything else I’ve heard.” Others reported actively checking various television sources in an attempt to gather bipartisan information:

When I watch the news, I don’t just watch one network … Because I know one’s supposed to be very liberal. One’s supposed to be very conservative. One’s supposedly pro-White House. So I try to take a little bit of each, so it will be more than one source.

Some participants used multiple subcategories of mass media to cross-reference information: “I wanted to get more information, so I did check other channels like NBC, ABC, and CNN. And also we get 2 daily newspapers.” Personal research became a part of an information-seeking routine for some: “When I hear something on CBS I kind of go out on the internet and see what the health organizations are saying about it, to kind of back up what I’m hearing.”

Besides traditional mass media sources, the internet offered a source for further research on emerging COVID-19 information: “I put in [Google] ‘What types of death qualifies as COVID deaths?’ I did actually find a CDC article that said that if someone dies with the COVID virus, it should be counted as a cause of death.” Personal research also included going to fact-checking websites: “I usually go to one of those fact-finding sites like Snopes.com. Usually they’re a pretty reliable source to find out whether something is an actual fact or if it’s false.” Participants often defended their use of internet sources as part of due diligence; as one said, “I know that sounds silly—‘on the internet.’ But I go to decent news sources. And when I do read them, I try to look up corroborating testimony.”

#### Confirmation Bias

Participants also exhibited confirmation bias in their decisions regarding what COVID-19 information to believe, choosing to trust information that matched their prior beliefs. Confirmation biases are generated endogenously from within as the individual draws from lived experience, relationships, the environment, and unique situations, in an attempt to determine the trustworthiness of emerging pieces of information.

Confirmation bias is often referred to as “common sense,” because it results from one’s lived experience. The lived experience of a lawsuit against a pharmaceutical company, for example, left 1 participant especially distrustful of COVID-19 vaccine makers: “I have a generalized distrust of pharmaceutical companies. They’re in it for the money, not to help people.” Common sense was also reflected in a prior understanding of how diseases spread indoors: “Yes [I trusted the information from CBS] because it made sense to me. Because we’re now gonna be indoors, it makes sense that’s what their cases look like [higher].” One participant demonstrated confirmation bias in discussing how to decide which information to trust and which to ignore:

I believe what I think is correct based on my past knowledge and experience from what I’ve seen from other sources and read in the newspapers. And ones in conflict with those, I just ignore.

Although some individuals gave the reasoning behind or specific examples of their lived experience, others stated their reliance on common sense proudly, without justification: “Partly I go by my gut—what strikes me as logical, reasonable, possible, probable.” One participant distrusted information in order to debunk the wisdom of wearing a mask: “I believe with my whole power of reasoning and my gut feeling and perceptions. I just didn’t buy it.” Another participant simply stated that information could be trusted “if it makes sense. Sometimes things don’t make sense, and I know they’re not true, so I just ignore it.” In explaining the trustworthiness of information from the cable network One American News, 1 participant said:

The first test is common sense, The second test is, does it square with my experience?...My experience and logic tells me that the people who are saying [hydroxychloroquine] doesn’t work, are people with an agenda.

Thus “common sense” could explain almost any position regarding what information should be trusted or believed.

#### Resigned Acceptance

The feeling that one has no other choice but to trust information that one is exposed to repeatedly, or a resigned acceptance of information, was another important criterion for trusting COVID-19 public health information. As an exogenous criterion, resigned acceptance consists of an outward reliance on repeated information as presented, without actively using strategies to assess its trustworthiness. For some, resigned acceptance relied on professional sources on television shows: “I would like to think that the information that’s provided on the television shows are truthful...they’re provided by professionals and I have no reason not to trust them.” The lack of knowledge was given as a reason for resigned acceptance of mass media: “Yes, I did trust the information [from Fox News] because I knew very little about it. And so naturally, I would acquiesce to what they were saying, and take as much as I possibly can because it was all foreign to me.”

Others thought that repeated corroborating information left them with no choice but to believe it: “I guess if you hear it over and over again from enough sources, you’re going to trust it. I don’t have anything else to go by.” Another participant began to pay attention to public health information from mass media only after hearing about repeated, increasingly deadly cases:

I really wasn’t paying attention to [the information from CNN] at first. They were saying in NYC all these people were dying. At first I said nah, it’s a bunch of baloney to that. But then you know, as days went on, weeks went on, months went on and it’s getting worse and worse and worse, I had to trust it.

### Information Sources, Trust, and Personal Criteria

Personal criteria for trusting COVID-19 public health information can be situated between a person’s trust in the source of the information and trust in the information itself. For example, personal relevance is invoked when information is trusted because the source of the information is more personally or socially proximate to the individual. Similarly, personal research is tied to trust in sources of information that the individual has sought in order to gather additional information, rejecting unreliable sources and accepting those that they trust. Personal relevance and personal research are based on trust in the information source. Yet personal relevance is an endogenous criterion, meaning that the trust is based on one’s own understanding or experience, whereas personal research is an exogenous criterion and garners trust from outside sources.

On the other hand, both confirmation bias and resigned acceptance are more closely tied to trust (or lack of trust) in particular pieces of information. Confirmation bias is seen when individuals automatically trust information because it confirms their preexisting beliefs, and resigned acceptance may be seen when individuals decide to accept information based on hearing it repeatedly from multiple sources. Confirmation bias is an endogenous criterion in that it relies on trust developed through preexisting beliefs, whereas resigned acceptance is an exogenous criterion because it relies on trust that comes from repeated exposure to external information sources.

## Discussion

### Principal Findings

In this interview study, we have identified sources that older adults turned to for trusted COVID-19 public health information and the factors that influenced their trust. We have also identified and examined 4 key criteria that influenced older adults’ trust in information sources for COVID-19 public health information. In Figure 1, we illustrate the relationships among these key criteria: the criteria are either endogenous or exogenous, and the trust in the information is based on either the source of the information or the information itself. Although other COVID-19 research has focused on individual criteria that influence trust, to the best of our knowledge, this is the first attempt to demonstrate the interplay among criteria.

The participants in this study relied on information sources such as mass media, known individuals, and the internet for COVID-19 public health information. The subcategories of individual sources that older adults trusted included media such as television news and newspapers; individuals such as friends and family; and internet sources such as websites maintained by authoritative health organizations, as well as search engines themselves. These information sources echo the findings of earlier work exploring older adults’ trust in COVID-19 public health information [6,20,31]. In addition, echoing the findings of earlier studies [12-14], participants reported using social media for social interactions and entertainment. However, the participants in our study did not trust social media for reliable information and adamantly refused to use social media to obtain COVID-19 public health information.

Prior research has examined how older adults consider the ways in which information is presented on the web and their resulting perceptions of trust [16]. The pandemic offers an opportunity to extend the current understanding of older adults’ trust and distrust of public health information during an unprecedented, fast-paced, and evolving public health crisis. Negative cognitive and emotional responses to COVID-19 have been found to decrease the integration of information from trusted sources and increase susceptibility to misinformation [5,21]. Participants in this study were perceptive to and leery of potential COVID-19 misinformation. They recognized that COVID-19 public health information changed quickly and was often contradictory. Although we did not use the word “misinformation” in our interview guide, we did ask participants to share a piece of information that they distrusted and where they had found it. Participants were adamant that the COVID-19 public health information found on social media was not trustworthy.

In total, 4 factors affected how older adults determined trust in information sources. First, participants indicated that the personal relevance of information was key to determining its reliability and trustworthiness. When a participant received information from a close social connection or witnessed it as physically or socially proximate, the participant was more likely to trust the information. Many spoke of trusting COVID-19 public health information from their local news, state health organizations, and others in their community, as well as of witnessing the pandemic’s effects themselves. This finding aligns with previous research in which individuals considered COVID-19 information most helpful when it was directly relevant to their own lives [31]. Chen et al [22] found that older adults weighed the trustworthiness of COVID-19 public health information on the basis of their evaluation of the source. As in our findings, older adults considered socially and physically proximate information to be more helpful and trustworthy [22,23].

Second, our participants relied on personal research to verify new information and determine its veracity. They reported different approaches to performing their own research on the trustworthiness of information. Some fact-checked multiple mass media sources against each other. Others used the internet to do research on information that they acquired from mass media. These findings echo prior research on trust in information [24], including research on older adults’ information-seeking during a pandemic. Moore and Hancock [25] found that, given the proper resources, older adults fact-checked more often than their younger counterparts did. They also found that older adults could combine modern information resources with their life experiences in judging the veracity of COVID-19 public health information. Although research has previously suggested that older adults are challenged by sifting through large quantities of health information on the web, the older adults in our study used web-based sources such as the World Health Organization (WHO) and Centers for Disease Control and Prevention (CDC) websites as a means to fact-check the information that they received from mass media. The older adults in our study also suggested that personal research was only 1 way to determine their trust in information.

Third, the older adults exhibited confirmation bias when they justified their trust in COVID-19 information, echoing previous studies [19,26]. Zhao et al [19] found that confirmation bias was an influencing factor in the propensity to share information. However, their sample did not consist of older adults exclusively, and the study focused on sharing health information via social media, which our participants actively avoided. Older adults tend to prefer health care providers as their main sources of health information while relying on friends and family to provide and interpret information from web-based health sources [6,15]. Similarly, the participants in our study relied on their direct personal contacts, such as friends and family, for trustworthy health information, and they were less likely to fact-check information given to them by direct personal contacts. Turner et al [15] suggested that older adults lacked confidence in assessing the quality of web-based health information, but our participants did not. A likely reason is that our participants were recruited from cloud platforms, so these older adults may have been more experienced with technology than the general older adult population or the sample in Turner et al [15].

Fourth, participants reported a resigned acceptance of COVID-19 public health information, reluctantly trusting it only after they had received it from multiple sources over time. The work of Unkelbach et al [17] on repeated exposure and repetition-induced truth indicates that the quantity of information and the hearing of the same information repeatedly over time affects individuals’ tendency to trust it. For our participants, repetition increased the perception of truth more than the information itself did. However, we also found that repeated exposure was only 1 factor. Both repeated exposure to information and confirmation bias were key to resigned acceptance of COVID-19 public health information. Our findings suggest that confirmation bias, the lack of viable alternate explanations, and repetitions of information all shape trust. The study of Unkelbach et al [17] was not focused on COVID-19 public health information, but the authors did discuss the implications of their findings within interventions to change false beliefs such as those of the antivaccination movement.

The results depicted in the semiotic square in Figure 1 reflect the dichotomy between trust focused on sources and trust focused on the information itself, as well as the relationships between them. Among the older adult participants in this study, personal relevance and confirmation bias were endogenous factors of trust; personal research and resigned acceptance were exogenous factors of trust. Simultaneously, personal relevance and personal research are factors in which older adults rely on their trust in particular sources of information, whereas confirmation bias and resigned acceptance depend on trust in the information itself. The evaluation of each of these criteria does not always occur independently; the criteria often overlap and influence each other. This complex interplay reflects the multifaceted nature of the personal and social factors in older adults’ navigation of COVID-19 public health information as they attempt to make sense of their world.

### Limitations and Future Directions

This study has some limitations. The study sample was from a pool of older adults for our earlier web-based survey study, so the results may not be representatively applicable to the general older adult population. Although Prime Panels allowed us to rapidly recruit and collect data from older adults (who might otherwise have been difficult to reach because of pandemic-related constraints [32]), older adults who use a web-based crowdsourcing platform may have higher digital literacy than those who do not. In addition, all but 1 of our research participants reported their race as White. Future work should focus on underrepresented subgroups of older adults.

It is critical to understand older adults’ trust in information during health crises in order to develop strategies for successful public health dissemination. In this CIT study, we have investigated older adults’ trust in new, rapidly changing public health information. A critical incident interviewing technique allowed participants to identify salient topics from which we could develop a framework for trust in COVID-19 public health information. Our qualitative approach ensured that participants’ own perspectives and experiences were represented without deductive, researcher-directed categories. However, future studies should also examine relationships between information sources and personal criteria using quantitative methods.

## Appendix

Multimedia Appendix 1 Interview Guide.

## Abbreviations

CDC: Centers for Disease Control and Prevention
CIT: critical incident technique
RQ: research question
WHO: World Health Organization
