# Supplementary material for: Older Adults’ Trust and Distrust in COVID-19 Public Health Information: Qualitative Critical Incident Study
Source: JMIR Aging. 2023 Nov 9;6:e42517. doi: 10.2196/42517 (PMC10637349; doi:10.2196/42517)
Supplement: Multimedia Appendix 1 [file aging_v6i1e42517_app1.docx]

Interview Guide

1. From what source did you first learn about COVID-19?

1. What was the information source? (Clarify source medium: Television, Facebook, and NewsFeed)
2. Did you trust the information you were hearing from this source? Why or why not?
3. How confident are you in the information from this source? Why?
4. What factors do you continue to use to assess the trustworthiness of that information?
5. Did this information change your day-to-day life in any way? Please explain.

2. What is the most recent health information you’ve heard about COVID-19?

1. What was the information source?
2. Did you trust the information? Why or why not?
3. How confident are you in this information? Why?
4. What factors do you continue to use to assess its trustworthiness?
5. Has this information changed your day-to-day life in any way? Please explain.

The next few questions are rather similar. I’m going to ask you for information you trust, information you don’t trust, information where your trust has changed, and information you just don’t know if you can trust. As I read the question, I’ll make sure to clearly state which type of information I’m asking about, but if you need clarification at any point, let me know. I’ll be happy to restate the question.

3. Please give me an example of COVID-19 health information that you **trust**.

1. Why did you trust this information?
2. What was the source of this information?
3. How confident are you in this information? Why?
4. Has your trust in this information changed over time?
5. Did the source of the information contribute to your trust of this information? (if not already answered)
6. Did this information change your day-to-day life in any way? Please explain. (if not already answered)

4. Please give me an example of COVID-19 health information that you **don’t** trust.

1. Why did you distrust this information?
2. What was the information source?
3. How confident are you in this information? Why?
4. Has your distrust in this information changed over time?
5. Did the source of the information contribute to your distrust of this information? (if not already answered)
6. Did this information change your day-to-day life in any way? Please explain. (if not already answered)

Alright, the next questions are similar. We’re going to talk about information where your trust has changed.

5. Please give me an example of COVID-19 health information that you **originally distrusted** but **now you do trust.**

1. Why did you originally distrust this information?
2. What led you to trust this information?
3. What was the information source and did that affect your trust or distrust? (if not already answered)
4. Did this information change your day-to-day life in any way? Please explain. (if not already answered)

6. Could you please give me an example of COVID-19 information that you **originally** **trusted** but now **do not trust**. (16 min)

1. Why did you originally trust this information?
2. What led you to distrust this information?
3. What was the information source and did that affect your trust or distrust? (if not already answered)
4. Did this information change your day-to-day life in any way? Please explain. (if not already answered)

7. Is there a piece of COVID-19-related health information that you’re not sure whether to trust or distrust? Tell me about it.

1. What factors lead you to trust this information?
2. What factors lead you to distrust this information?
3. What was the information source and how does that affect your trust or distrust? (if not already answered)
4. How would trusting or distrusting this information change your day-to-day life? Please explain. (if not already answered)

8. Do you use social media? (If yes, continue; if no, prompt with examples) Examples, if needed: Facebook, Twitter, Instagram, and WhatsApp

1. When you encounter COVID-19 health information on social media, do you normally pay attention to who posted it?

i. Does that affect how you perceive that information?

1. How has interacting with COVID-19 information on social media affected your relationships?
2. Have any conflicts with others arisen from this?

i. If, so how have you managed that?

1. When you consider sharing COVID-19 health information on social media, how do the potential reactions of individuals in your social network influence your decision to share?
2. When you think about sharing COVID-19 information, do you think about how others might change their perceptions of you because of the information that you shared?
3. Have any groups that you are involved with on social media influenced your views on COVID-19? Please explain.
4. How have your personal beliefs and experiences influenced how you interact with COVID-19 content on social media?
